# Supplementary material for: Time‑resolved multi-omic analysis of paclitaxel exposure in human iPSC‑derived sensory neurons unveils mechanisms of chemotherapy‑induced peripheral neuropathy
Source: Cell Death Dis. 2026 Feb 10;17(1):211. doi: 10.1038/s41419-026-08445-2 (PMC12921266; doi:10.1038/s41419-026-08445-2)
Supplement: Supplementary file 15 — Dataset 1. [file 41419_2026_8445_MOESM15_ESM.zip › CDD_Open_Data/04_RNA_Seq/DE_all_and_otherCL_20241209.tar.gz!/DE/all/pipeline_report/out/pipeline_report.all.report.html]

xml version="1.0" encoding="utf-8"?


untitled Docutils document


System Message: ERROR/3 ()

Line 45 exceeds the line-length-limit.
